# Externally controlled high degree of spin polarization and spin inversion in a conducting junction: Two new approaches

**DOI:** 10.1038/s41598-017-14499-2

**Published:** 2017-10-30

**Authors:** Moumita Patra, Santanu K. Maiti

**Affiliations:** Physics and Applied Mathematics Unit, Indian Statistical Institute, 203 Barrackpore Trunk Road, Kolkata, 700 108 India

## Abstract

We propose two new approaches for regulating spin polarization and spin inversion in a conducting junction within a tight-binding framework based on wave-guide theory. The system comprises a magnetic quantum ring with finite modulation in site potential is coupled to two non-magnetic electrodes. Due to close proximity an additional tunneling is established between the electrodes which regulates electronic transmission significantly. At the same time the phase associated with site potential, which can be tuned externally yields controlled transmission probabilities. Our results are valid for a wide range of parameter values which demonstrates the robustness of our proposition. We strongly believe that the proposed model can be realized in the laboratory.

## Introduction

The way of getting selective spin transmission through a conducting junction has always been an interesting topic in the subject of spintronics^1,2^. The most common route of generating polarized spin currents is the use of ferromagnetic electrodes^3,4^ though it has strong limitations due to resistivity mismatch^5^. Utilizing a simple quantum dot (QD) driven by radio frequency gate voltages one can also get polarized spin current in presence of moderate in-plane magnetic field^6,7^.

For purposeful design of spintronics devices like spin filters, spin transistors, single spin memories, solid state qubits, etc., the generation of polarized spin current is not the only requirement, but its proper regulation is highly significant^8–10^. Some intrinsic properties, for example, spin-orbit (SO) interaction which couples electron’s spin to the charge degree of freedom provides deeper insight^11–15^ for generating polarized spin current. Usually two types of SO interactions, namely Rashba^16^ and Dresselhaus^17^, are encountered in solid state materials, out of which Rashba SO coupling, originated from the lacking of structural symmetry, plays the key role for selective spin transfer as one can regulate its coupling strength by external gate potential^18,19^.

For the *three-terminal case* where a bridging material is connected with three electrodes this approach is highly appreciated^20–23^. Whereas for the *two-terminal system* only SO coupling is not capable for producing polarized spin currents as it does not break the Kramer’s degeneracy between $|k\uparrow \rangle$ and $|-k\downarrow \rangle$ states^24,25^. Thus one has to incorporate magnetic impurities or magnetic field to achieve this goal^26^ which essentially brings the difficulty as confining a strong magnetic field in a nano-scale region such as quantum dot or nano-ring is not so trivial.

Few other approaches have also been discussed to achieve higher degree of spin polarization. For instance, an organic polymer coupled to a quantum wire can exhibit selective spin transmission^27^ where the spin polarization is manipulated by an external gate voltage, instead of external magnetic field. In another work, Lindelof *et al*., have proposed^28^ spin reversal in a QD coupled to ferromagnetic leads by purely electrical means which provides the fundamental importance of designing spintronics devices. Recently one of the authors of us has also shown that controlled spin dependent transport can be obtained^29^ through a magnetic quantum wire coupled to a magnetic quantum ring in presence of in-plane electric field. This in-plane electric field regulates electronic transport through the junction in a controlled way.

Till date many works have been done both theoretically as well as experimentally and have already revealed several unique features^27–44^ of spin selective transmission. *But very less amount of these works have discussed the fact of externally controlled selective spin transfer through a nano-junction which is highly significant in designing controlled spintronics devices*. This essentially motivates us, and in the present work we intend to explore a possible route of getting externally controlled spin dependent transport.

**Figure 1:**
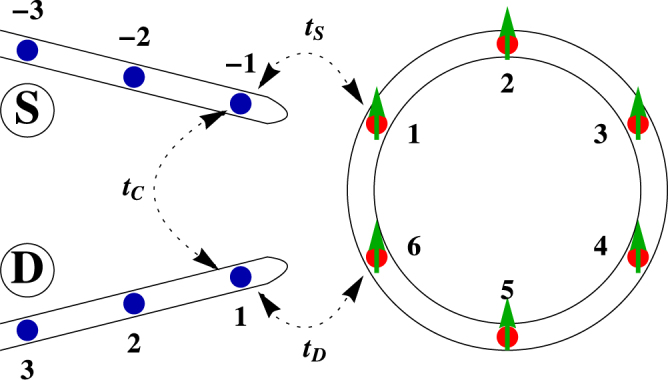
Schematic view of conducting nano-junction where a magnetic quantum ring with continuous modulation in site energy is coupled to two non-magnetic electrodes. Due to close proximity an additional *new path* is established between source (S) and drain (D) electrodes, which is one of the key control parameters of our study. Filled colored circles correspond to the atomic sites where magnetic atoms, having a finite magnetic moment, are trapped. The direction of the magnetic moment in each site is described by the green arrow.

We consider a simple two-terminal junction, where the bridging system is a magnetic quantum ring. A finite modulation in site energy (described by ${\varepsilon }_{i}$, $i$ being the site index) is given in the form of Aubry-André-Harper (AAH) model^45–47^ i.e., ${\varepsilon }_{i}=w\,\cos \,\mathrm{(2}i\pi \lambda +{{\varphi }}_{\nu })$, where $w$ describe the width of the site energy, and $\lambda$ is an irrational number which is fixed at $\mathrm{(1}+\sqrt{5}\mathrm{)/2}$ (golden mean). The phase factor ${{\varphi }}_{\nu }$ associated with this expression plays an important role to regulate electron transmission, more precisely, spin transmission. This ${{\varphi }}_{\nu }$ *can be tuned externally*, which thus suggests a possible route of regulating spin transmission, without directly disturbing any other physical parameters. At the end of our theoretical analysis the feasibility of implementing such a model in laboratory is discussed. Along with this, we propose another way of current regulation by introducing the *proximity effect* of two non-magnetic source and drain electrodes those are coupled to the neighboring sites of the ring (see Fig. 1). Due to close proximity an additional coupling is established between the end atomic sites of the electrodes so that electrons can directly tunnel between them^48,49^ including their propagation through the magnetic quantum ring. This coupling which is of course tunable, plays a significant role in current regulation. From our numerical results we see that the present model exhibits a very high degree of spin polarization, some cases it almost reaches to $\mathrm{100 \% }$ and at the same time complete spin reversal can be achieved. Our results are valid for a wide range of parameter values, which demonstrates the robustness of our proposition, and we strongly believe that both the two approaches can be implemented experimentally.

## Molecular Model and Theoretical Framework

### Model and Hamiltonian

Let us begin with the nano-junction shown in Fig. 1 where a $N$-site magnetic quantum ring is coupled to two perfect non-magnetic semi-infinite metallic electrodes, namely, source and drain. Each site of the ring is accompanied with a local magnetic moment with amplitude ${h}_{i}$ and its orientation is described by the polar angle ${\theta }_{i}$ and azimuthal angel ${{\phi }}_{i}$ in spherical polar coordinate system. At the same time, the site energies get modified following the relation $w\,\cos \,\mathrm{(2}i\pi \lambda +{{\varphi }}_{\nu })$ i.e., in the form of famous AAH model. Thus the bridging material is essentially a *correlated disordered system*, where the disorder is introduced only in site energy (viz, diagonal correlated disordered model).

On the other hand, the two site-attached electrodes are perfect as well as non-magnetic. Due to close proximity a direct coupling, described by the parameter ${t}_{C}$, exists between the two end atomic sites of the electrodes. This strength can be regulated either by changing the separation between the electrodes or by rotating them^49^.

In order to write the Hamiltonian of the nano-junction we use Tight-Binding (TB) framework which is extremely suitable for analyzing electron transport particularly in the absence of electron-electron interaction. Within the nearest-neighbor hopping approximation the Hamiltonian of the full system looks like

1 $${\bf{H}}={{\bf{H}}}_{{\bf{R}}}+{{\bf{H}}}_{{\bf{S}}}+{{\bf{H}}}_{{\bf{D}}}+{{\bf{H}}}_{{\bf{T}}}$$

where different sub-Hamiltonians correspond to different parts as described below. The Hamiltonian of the magnetic quantum ring is written as^31,33,40^

2 $${{\bf{H}}}_{{\bf{R}}}=\sum _{i}{{\boldsymbol{c}}}_{{\boldsymbol{i}}}^{{\boldsymbol{\dagger }}}({{\boldsymbol{\epsilon }}}_{{\boldsymbol{i}}}{\boldsymbol{-}}{{\boldsymbol{h}}}_{{\boldsymbol{i}}}\,\mathrm{.}{\boldsymbol{\sigma }}\,)\,{{\boldsymbol{c}}}_{{\boldsymbol{i}}}+\sum _{i}({{\boldsymbol{c}}}_{{\boldsymbol{i}}{\boldsymbol{+}}1}^{{\boldsymbol{\dagger }}}{{\boldsymbol{t}}}_{{\boldsymbol{i}}}{{\boldsymbol{c}}}_{{\boldsymbol{i}}}+h\mathrm{.}c\mathrm{.})$$

where,

$${{\boldsymbol{c}}}_{{\boldsymbol{i}}}\,=(\begin{array}{c}{{\bf{c}}}_{{\bf{i}}{\boldsymbol{\uparrow }}}\\ {c}_{i\downarrow }\end{array}),\,{{\boldsymbol{c}}}_{{\boldsymbol{i}}}^{{\boldsymbol{\dagger }}}\,=(\begin{array}{cc}{c}_{i\uparrow }^{\dagger } & {c}_{i\downarrow }^{\dagger }\end{array}),{{\boldsymbol{t}}}_{{\boldsymbol{i}}}=(\begin{array}{cc}t & 0\\ 0 & t\end{array}),{{\boldsymbol{\epsilon }}}_{{\boldsymbol{i}}}=(\begin{array}{cc}{\epsilon }_{i} & 0\\ 0 & {\epsilon }_{i}\end{array}),{{\boldsymbol{h}}}_{{\boldsymbol{i}}}\mathrm{.}{\boldsymbol{\sigma }}={h}_{i}(\begin{array}{cc}\cos \,{\theta }_{i} & \sin \,{\theta }_{i}{e}^{-j{\phi }_{i}}\\ \sin \,{\theta }_{i}{e}^{j{\phi }_{i}} & -\cos \,{\theta }_{i}\end{array}).$$

Here $t$ and ${\varepsilon }_{i}$ correspond to the nearest-neighbor hopping (NNH) integral and site energy, respectively, in the ring. This site energy (${\varepsilon }_{i}$) is taken in the form of diagonal AAH model as discussed above. The term ${{\bf{h}}}_{{\bf{i}}}\mathrm{.}\,{\boldsymbol{\sigma }}$ describes the interaction of injected electron with the local magnetic moment placed at $i$-th site having strength ${h}_{i}$. $\,{\boldsymbol{\sigma }}\{={{\boldsymbol{\sigma }}}_{x},{{\boldsymbol{\sigma }}}_{y},\,{{\boldsymbol{\sigma }}}_{z}\}$ denotes the Pauli spin matrices in ***σ***_***z***_ diagonal representation.

The second and third sub-Hamiltonians in the right side of Eq. 1 represent the source and drain electrodes, and they are expressed as

3 $${{\bf{H}}}_{{\bf{S}}}=\sum _{n\le -1}{{\bf{a}}}_{{\bf{n}}}^{\dagger }\,{{\boldsymbol{\epsilon }}}_{{\bf{0}}}{{\bf{a}}}_{{\bf{n}}}+\sum _{n\le -1}({{\bf{a}}}_{{\bf{n}}}^{\dagger }{{\bf{t}}}_{{\bf{0}}}{{\bf{a}}}_{{\bf{n}}-{\bf{1}}}+h\mathrm{.}c\mathrm{.})$$

and

4 $${{\bf{H}}}_{{\bf{D}}}=\sum _{n\ge 1}{{\bf{b}}}_{{\bf{n}}}^{\dagger }\,{{\boldsymbol{\epsilon }}}_{{\bf{0}}}{{\bf{b}}}_{{\bf{n}}}+\sum _{n\ge 1}({{\bf{b}}}_{{\bf{n}}}^{\dagger }{{\bf{t}}}_{{\bf{0}}}{{\bf{b}}}_{{\bf{n}}+{\bf{1}}}+h\mathrm{.}c\mathrm{.})$$

where ${a}_{n}({b}_{n})$ and ${a}_{n}^{\dagger }({b}_{n}^{\dagger })$ are the annihilation and creation operators, respectively, for the source (drain) electrode. The other symbols are

$$\,{{\boldsymbol{\epsilon }}}_{{\bf{0}}}=(\begin{array}{cc}{\epsilon }_{0} & 0\\ 0 & {\epsilon }_{0}\end{array}),{{\boldsymbol{t}}}_{{\boldsymbol{0}}}=(\begin{array}{cc}{t}_{0} & 0\\ 0 & {t}_{0}\end{array}),$$

where ${\epsilon }_{0}$ and ${t}_{0}$ are the site-energy and nearest-neighbor hopping integral in the electrodes, respectively.

Finally, ${{\bf{H}}}_{{\bf{T}}}$, the tunneling Hamiltonian can be written as,

5 $${{\bf{H}}}_{{\bf{T}}}=({{\bf{c}}}_{{\bf{1}}}^{\dagger }{{\bf{t}}}_{{\bf{S}}}{{\bf{a}}}_{-{\bf{1}}}+{{\bf{c}}}_{{\bf{N}}}^{\dagger }{{\bf{t}}}_{{\bf{D}}}{{\bf{b}}}_{{\bf{1}}}+{{\bf{a}}}_{-{\bf{1}}}^{\dagger }{{\bf{t}}}_{{\bf{C}}}{{\bf{b}}}_{{\bf{1}}}+h\mathrm{.}c\mathrm{.})$$

where,

$$\,{{\bf{t}}}_{{\bf{K}}}=(\begin{array}{cc}{t}_{K} & 0\\ 0 & {t}_{K}\end{array})\,,K=S,D,C.$$

Here, ${t}_{S}$ and ${t}_{D}$ describe the couplings of the ring with source and drain, respectively and ${t}_{C}$ measures the direct coupling between the end atomic sites of the electrodes.

Below we discuss the theoretical prescription which includes the calculations of spin dependent transmission probabilities, junction currents and spin polarization.

### Transmission Probability

To calculate transmission probabilities we use wave-guide theory (which is very simple to understand)^48–51^. *The theoretical prescription given below is an extension of earlier studies where spin degrees of freedom have not been taken into account*. Here we consider electron spin and the required steps are as follows.

Let us start with the station wave-function of the entire system (viz, source-ring-drain)

6 $$|\psi \rangle =[\sum _{n\le -1}{{\bf{A}}}_{{\bf{n}}}\,{{\bf{a}}}_{{\bf{n}},{\boldsymbol{\sigma }}}^{\dagger }\,+\sum _{n\ge 1}{{\bf{B}}}_{{\bf{n}}}\,{{\bf{b}}}_{{\bf{n}},{\boldsymbol{\sigma }}}^{\dagger }\,+\sum _{i=1}\,{{\bf{C}}}_{{\bf{i}}}{{\bf{c}}}_{{\bf{i}},{\boldsymbol{\sigma }}}^{{\boldsymbol{\dagger }}}]\mathrm{|0}\rangle $$

where,

$${{\bf{A}}}_{{\bf{n}}}=(\begin{array}{c}{A}_{n,\uparrow }\\ {A}_{n,\downarrow }\end{array}),{{\bf{B}}}_{{\bf{n}}}=(\begin{array}{c}{B}_{n,\uparrow }\\ {B}_{n,\downarrow }\end{array}),{\rm{and}}\,{{\bf{C}}}_{{\bf{n}}}=(\begin{array}{c}{C}_{n,\uparrow }\\ {C}_{n,\downarrow }\end{array})$$

The coefficients ${A}_{n,\sigma }$, ${B}_{n,\sigma }$, and ${C}_{n,\sigma }$ correspond to the amplitude for an electron having spin $\sigma$ ($\uparrow$ or $\downarrow$) at the $n\,$ th site of the source, drain, and $i\,$ th site of the ring, respectively.

With this wave function we can write a set of coupled linear equations from the time-independent Schrödinger equation ${\bf{H}}|\psi \rangle$ $=$ ${E}{\bf{I}}|\psi \rangle$ (${\bf{I}}$ being the ($2\times 2$) identity matrix) as:

7 $$\begin{array}{rcl}(E{{\bf{I}}}_{{\bf{2}}}-{{\boldsymbol{\epsilon }}}_{{\bf{0,}}{\boldsymbol{\sigma }}}){{\bf{A}}}_{{\bf{n}}} & = & {{\boldsymbol{t}}}_{{\boldsymbol{0,}}{\boldsymbol{\sigma }}}({{\bf{A}}}_{{\bf{n}}+{\bf{1}}}+{{\bf{A}}}_{{\bf{n}}-{\bf{1}}}),n\le -\mathrm{2,}\\ (E{{\bf{I}}}_{{\bf{2}}}-{{\boldsymbol{\epsilon }}}_{{\bf{0,}}{\boldsymbol{\sigma }}}){{\bf{A}}}_{-{\bf{1}}} & = & \,{{\boldsymbol{t}}}_{{\boldsymbol{0,}}{\boldsymbol{\sigma }}}{{\bf{A}}}_{-{\bf{2}}}+{{\boldsymbol{t}}}_{{\boldsymbol{C}},{\boldsymbol{\sigma }}}{{\bf{B}}}_{{\bf{1}}}+{{\boldsymbol{t}}}_{S{\boldsymbol{,}}{\boldsymbol{\sigma }}}{{\bf{C}}}_{{\bf{1}}},\\ (E{{\bf{I}}}_{{\bf{2}}}-{{\boldsymbol{\epsilon }}}_{{\bf{0,}}{\boldsymbol{\sigma }}}){{\bf{B}}}_{{\bf{n}}} & = & {{\boldsymbol{t}}}_{{\boldsymbol{0,}}{\boldsymbol{\sigma }}}({{\bf{B}}}_{{\bf{n}}+{\bf{1}}}+{{\bf{B}}}_{{\bf{n}}-{\bf{1}}}),n\ge \mathrm{2,}\\ (E{{\bf{I}}}_{{\bf{2}}}-{{\boldsymbol{\epsilon }}}_{{\bf{0,}}{\boldsymbol{\sigma }}}){{\bf{B}}}_{{\bf{1}}} & = & {{\boldsymbol{t}}}_{{\boldsymbol{0,}}{\boldsymbol{\sigma }}}{{\bf{B}}}_{{\bf{2}}}+{{\boldsymbol{t}}}_{{\boldsymbol{C}},{\boldsymbol{\sigma }}}{{\bf{A}}}_{-{\bf{1}}}+{{\boldsymbol{t}}}_{D{\boldsymbol{,}}{\boldsymbol{\sigma }}}{{\bf{C}}}_{{\bf{N}}},\\ (E{{\bf{I}}}_{{\bf{2}}}-{{\boldsymbol{\epsilon }}}_{{\boldsymbol{i}}{\boldsymbol{,}}{\boldsymbol{\sigma }}}){{\bf{C}}}_{{\bf{i}}} & = & {{\boldsymbol{t}}}_{{\boldsymbol{i}},{\boldsymbol{\sigma }}}({{\bf{C}}}_{{\bf{i}}+{\bf{1}}}+{{\bf{C}}}_{{\bf{i}}-{\bf{1}}})+{{\boldsymbol{t}}}_{S{\boldsymbol{,}}{\boldsymbol{\sigma }}}{\delta }_{i\mathrm{,1}}{{\bf{A}}}_{-{\bf{1}}}\\  &  & +{{\boldsymbol{t}}}_{D{\boldsymbol{,}}{\boldsymbol{\sigma }}}{\delta }_{i,N}{{\bf{B}}}_{{\bf{1}}},1\le i\le N\end{array}$$

#### Up spin incidence from the source lead

Assuming a plane wave incidence for up spin electrons with unit amplitude, we can write the amplitudes as:

$${{\boldsymbol{A}}}_{{\boldsymbol{n}}}=(\begin{array}{c}{e}^{ik(n+\mathrm{1)}a}+{r}_{\uparrow \uparrow }{e}^{-ik(n+\mathrm{1)}a}\\ {r}_{\uparrow \downarrow }{e}^{-ik(n+\mathrm{1)}a}\end{array})\,\mathrm{and}\,{{\boldsymbol{B}}}_{{\boldsymbol{n}}}=(\begin{array}{c}{t}_{\uparrow \uparrow }{e}^{ikna}\\ {t}_{\uparrow \downarrow }{e}^{ikna}\end{array}),$$

where $a$ being the lattice spacing and $k$ is the wave vector associated with the energy $E$. The other parameters are as follows: ${t}_{\uparrow \uparrow }$ = Transmission amplitude of a up spin ($\uparrow$) transmitted as up spin ($\uparrow$),

${t}_{\uparrow \downarrow }$ = Transmission amplitude of a up spin ($\uparrow$) transmitted as down spin ($\downarrow$).

${r}_{\uparrow \uparrow }$ = Reflection amplitude of a up spin ($\uparrow$) reflected as up spin ($\uparrow$),

${r}_{\uparrow \downarrow }$ = Reflection amplitude of a up spin ($\uparrow$) reflected as down spin ($\downarrow$).

Using the expression of ${{\bf{A}}}_{{\bf{n}}}$ and ${{\bf{B}}}_{{\bf{n}}}$ we can now find the reflection and transmission amplitudes by solving the set of coupled equations (Eq. 7) for a particular energy associated with each wave vector $k\,$. The we can define the pure spin transmission and spin flip transmission probabilities as ${T}_{\uparrow \uparrow }=|{t}_{\uparrow \uparrow }{|}^{2}$ and ${T}_{\uparrow \downarrow }=|{t}_{\uparrow \downarrow }{|}^{2}$, respectively for the case of up spin incidence.

#### Down spin incidence from the source lead

For the case of down spin incidence the amplitudes ${{\bf{A}}}_{{\bf{n}}}$ and ${{\bf{B}}}_{{\bf{n}}}$ look like:

$${{\bf{A}}}_{{\bf{n}}}=(\begin{array}{c}{r}_{\downarrow \uparrow }{e}^{-ik(n+\mathrm{1)}a}\\ {e}^{ik(n+\mathrm{1)}a}+{r}_{\downarrow \downarrow }{e}^{-ik(n+\mathrm{1)}a}\end{array})\,{\rm{and}}\,{{\bf{B}}}_{{\bf{n}}}=(\begin{array}{c}{t}_{\downarrow \uparrow }{e}^{ikna}\\ {t}_{\downarrow \downarrow }{e}^{ikna}\end{array}),$$

where the meaning of different factors are as follows:

${t}_{\downarrow \uparrow }$ = Transmission amplitude for down spin ($\downarrow$) transmitted as up spin ($\uparrow$),

${t}_{\downarrow \downarrow }$ = Transmission amplitude for down spin ($\downarrow$) transmitted as down spin ($\downarrow$).

${r}_{\downarrow \uparrow }$ = Reflection amplitude for down spin ($\downarrow$) reflected as up spin ($\uparrow$),

${r}_{\downarrow \downarrow }$ = Reflection amplitude for down spin ($\downarrow$) reflected as down spin ($\downarrow$).

Using the same prescription as stated for the case of up spin incidence, here also we can calculate all coefficients by solving the equations given in Eq. 7, and eventually, find the transmission probabilities as ${T}_{\downarrow \downarrow }=|{t}_{\downarrow \downarrow }{|}^{2}$ and ${T}_{\downarrow \uparrow }=|{t}_{\downarrow \uparrow }{|}^{2}$.

Finally we can write the total transmission probability for spin up as ${T}_{\uparrow }={T}_{\uparrow \uparrow }+{T}_{\downarrow \uparrow }$ and for spin down as ${T}_{\downarrow }={T}_{\uparrow \downarrow }+{T}_{\downarrow \downarrow }$.

### Junction Current

Once the transmission function is determined, the net junction current for a particular bias voltage $V$ at absolute zero temperature, can be evaluated from the relation^52^

8 $${I}_{\sigma }(V)=\frac{e}{h}{\int }_{{E}_{F}-\frac{eV}{2}}^{{E}_{F}+\frac{eV}{2}}{T}_{\sigma }(E)\,dE$$

where ${E}_{F}$ is the equilibrium Fermi energy.

### Spin Polarization

Finally, we define spin polarization coefficient as^53^

9 $$P=\frac{{I}_{\uparrow }-{I}_{\downarrow }}{{I}_{\uparrow }+{I}_{\downarrow }}$$

*P* = +1(−1) corresponds to the only up (down) spin propagation, and thus, under this situation the degree of up (down) spin polarization becomes $\mathrm{100 \% }$. $P=0$ represents no spin polarization.

## Numerical Results and Discussion

Following the above theoretical prescription now we present our numerical results. The physical parameters those are kept constant throughout the computation are as follows. In the source and drain electrodes, the site energy ${\epsilon }_{0}$ and nearest-neighbor hopping integral ${t}_{0}$ are fixed at $0$ and $3\,$ eV, respectively, whereas in the bridging conductor (i.e., the ring) we set $t=1\,$ eV and choose ${\epsilon }_{i}$ following the relation ${\epsilon }_{i}=w\,\cos (2i\pi \lambda +{{\varphi }}_{\nu })$ considering $w=1\,$ eV. In the ring conductor we consider the strength of magnetic moment ${h}_{i}=1\,$ eV and the azimuthal angle ${{\phi }}_{i}=0$ for all $i$ and also, unless otherwise specified, ${\theta }_{i}=0$ for all $i$. The other two parameters ${t}_{S}$ and ${t}_{D}$ are fixed at $1\,$ eV. The values of ${t}_{C}$ and phase factor ${{\varphi }}_{\nu }$ are placed in appropriate figures, as they are not constant. All the calculations presented below are computed at absolute zero temperature setting equilibrium Fermi energy ${E}_{F}=0$.

**Figure 2:**
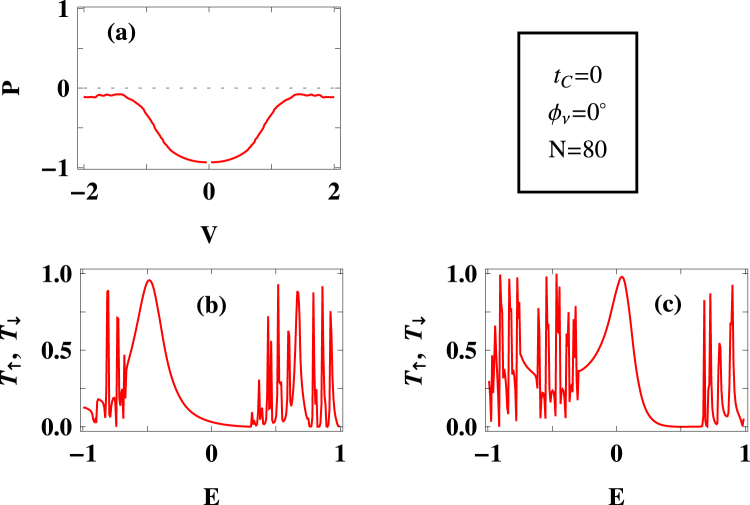
Voltage dependent spin polarization coefficient $P$ along with spin dependent transmission probabilities ${T}_{\uparrow }$ and ${T}_{\downarrow }$ as a function of injecting electron energy $E$ for a $80$-site ring at some typical values of ${{\varphi }}_{\nu }$ and ${t}_{C}$. At zero bias ($V=0$) there is no current across the junction, and thus, we cannot take the ratio of the currents following Eq. 9 as it is undefined. Therefore, we ignore this point in the $P$-$V$ curve.

**Figure 3:**
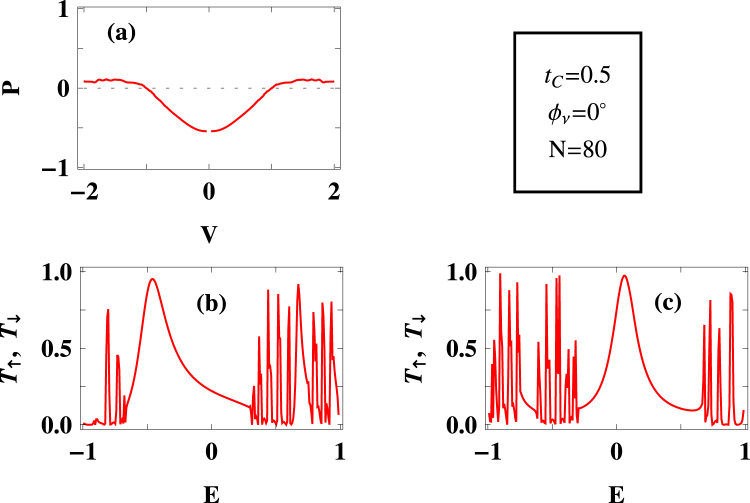
Same as Fig. 2, with ${t}_{C}=0.5\,$ eV.

**Figure 4:**
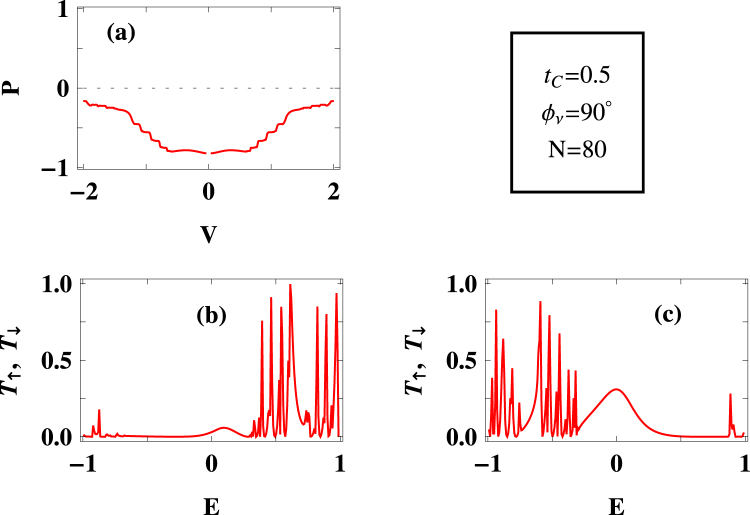
Same as Fig. 2, with ${t}_{C}=0.5\,$ eV and ${{\varphi }}_{\nu }=\pi \mathrm{/2}$.

Before addressing the central issues i.e., regulations of spin polarization as well as spin inversion with the help of external phase ${{\varphi }}_{\nu }$ and direct coupling parameter ${t}_{C}$, let us start by analyzing spin polarization coefficient for some typical values of ${t}_{C}$ and ${{\varphi }}_{\nu }$. The results are presented in Figs 2–4, where the variation of spin polarization $P$ is given as a function of bias voltage $V$ along with up and down spin transmission probabilities considering the ring size $N=80$.

For ${t}_{C}=0$ and ${{\varphi }}_{\nu }=0\,$, the spin polarization coefficient $P$ almost reaches to a maximum for low bias region (*P* = +1 or P = −1 represents a maximum spin polarization associated with the complete suppression of down or up spin propagation through the junction), and it ($P$) gradually decreases with increasing bias voltage and eventually drops almost to zero for higher voltages (Fig. 2(a)). This behavior can be justified from the transmission spectra given in Fig. 2(b) and (c). For narrow energy window across $E=0\,$, transmission probability of up spin electrons is almost zero whereas finite transmission of other spin electrons is obtained which results $P\sim -1$ over a narrow voltage region associated with the energy window. But when we consider wide energy region, associated with the bias voltage, both up and down spin channels contribute in electronic transmissions yielding lesser spin polarization.

The spin polarization, more precisely spin selective transmission, essentially depends on the separation between up and down spin channels. For such a system, where atomic sites of the bridging conductor are magnetic, spin flip interaction term is responsible for it. As hopping integral is fixed (same for both up and down spin electrons), the separation between the up and down spin channels is controlled by the term $({\varepsilon }_{i}-{h}_{i}\mathrm{.}\sigma )\,$, out of which ${\varepsilon }_{i}$ again contains a tunable factor ${{\varphi }}_{\nu }$ and its precise role can be understood from the forthcoming analysis.

Apart from this factor (i.e., ${\varepsilon }_{i}-{h}_{i}\mathrm{.}\sigma \,$), quantum interference has significant role on spin selective transmission. To reveal this fact let us focus on the results placed in Fig. 3(a), where we set a finite ${t}_{C}\,$, keeping all other parameters unchanged as taken in Fig. 2(a). Introduction of ${t}_{C}$ means there is an addition of a new path along with two conducting paths (namely longer and shorter paths in the magnetic quantum ring). Thus, these three paths are responsible for electronic transmission and we get the combined effect in the drain electrode. In presence of ${t}_{C}$ the degree of spin polarization gets reduced, compared to the previous case (viz, Fig. 2(a)), which is clearly noticeable in the low bias region (Fig. 3(a)). This reduction of spin polarization is expected because of the inclusion of new path which allows in certain percentage to pass up and down spin electrons, avoiding the magnetic ring. This is reflected in the transmission-energy spectra where we get finite transmission probabilities for both up and down spin electrons. So for a particular voltage window both of them are contributing, and depending on the contributing electrons we get a net polarization (which of course is less than $100\, \%$). For large enough ${t}_{C}$, one can expect much lesser spin polarization for any bias window as in that case electrons directly pass through this new path, without encountering any spin dependent interaction in the magnetic ring.

Under this situation if we incorporate the phase factor ${{\varphi }}_{\nu }$ then transmission spectra for both up and down spin electrons get modified, (Fig. 4(b) and (c)), and accordingly, spin polarization changes (Fig. 4(a)). Around $80\, \%$ spin polarization is achieved for a wide bias window, though eventually it decreases with higher voltages like the other two cases (viz, Figs 2 and 3).

From the results analyzed so far (i.e., Figs 2–4), we see that in the low bias region down spin electrons dominate suppressing the other spin electrons. An exactly opposite behavior might be observed for other set of parameter values depending on the channel separation, which in principle, is regulated by several factors for the present model.

### Regulation of spin polarization by ${t}_{C}$

**Figure 5:**
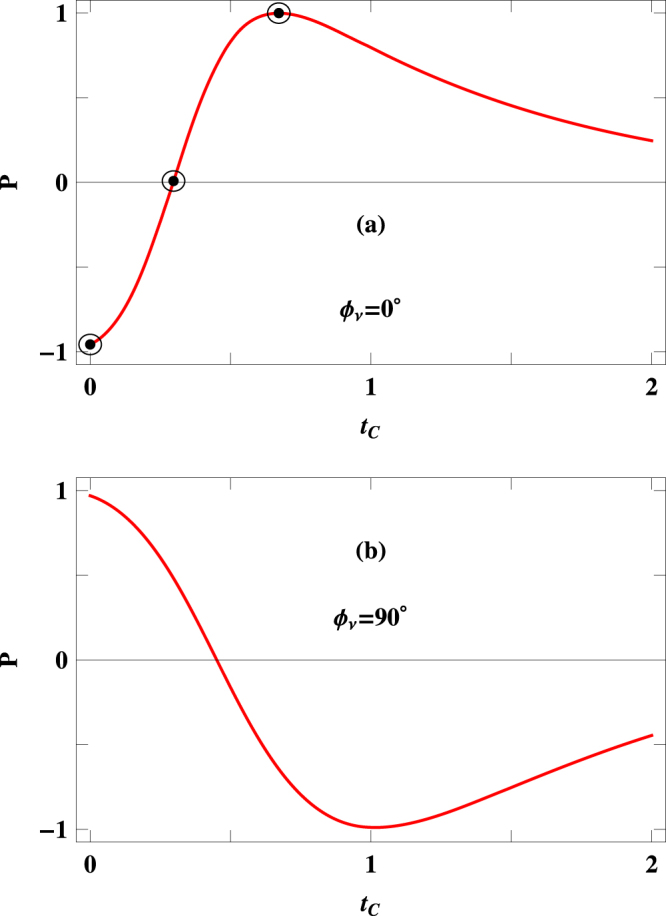
Spin polarization coefficient $P$ as a function of ${t}_{C}$ for a $120$-site ring considering two different values of ${{\varphi }}_{\nu }$. Here we set $V=0.25\,$ V.

Now we discuss the explicit dependence of spin polarization $P$ on the coupling parameter ${t}_{C}$. The results are presented in Fig. 5 for a $120$-site ring considering two different values of ${{\varphi }}_{\nu }$. Two observations are noteworthy. First, by regulating the external tunneling coupling ${t}_{C}$, $P$ can be changed widely from $+1$ to $-1$ and vice versa. Second, a phase reversal of spin polarization takes place with the help of AAH phase ${{\varphi }}_{\nu }$. When ${{\varphi }}_{\nu }=0$, $P$ varies from $-1$ to $+1$, while for the other case (${{\varphi }}_{\nu }=\pi \mathrm{/2}$), it ($P$) runs from $+1$ to $-1$, and for large ${t}_{C}$ decreasing spin polarization is observed in these two cases.

**Figure 6:**
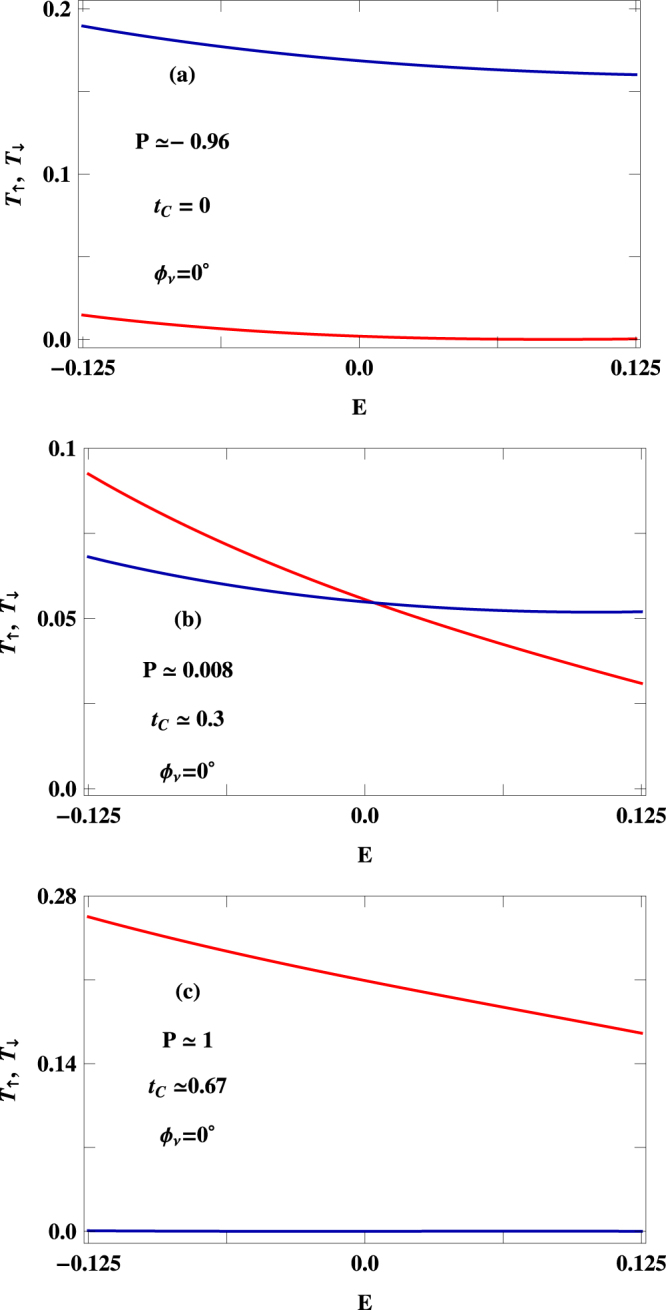
Energy dependence of ${T}_{\uparrow }$ (red curve) and ${T}_{\downarrow }$ (blue curve) at three different values ${t}_{C}$ those are represented by encircled dots in Fig. 5(a). The other physical parameters are same as taken in Fig. 5.

To implement this wide variation of $P$, we choose three distinct points from $P$-${t}_{C}$ curve of Fig. 5(a), represented by encircled dots, and present the characteristics of up and down spin transmission probabilities for these ${t}_{C}$ in Fig. 6. The results are shown for a specific energy window (−$0.125\le E\le 0.125$) associated with the voltage $V=0.25\,$ V. When ${{\varphi }}_{\nu }=0$ and ${t}_{C}=0$, up spin transmission probability is almost zero (red line of Fig. 6(a)), while finite transmission probability is obtained for down spin electrons (blue line of Fig. 6(a)) which results $P\sim -1$. The scenario gets reversed at ${t}_{C}\simeq 0.67$, shown in Fig. 6(c), where only up spin electrons transmit through the junction providing *P* = +1. At ${t}_{C}\simeq 0.3$, finite transmission probabilities are obtained for both up and down spin electrons, and ${I}_{\uparrow }$ is very close to ${I}_{\downarrow }$ which gives vanishing spin polarization (Fig. 6(b)). Similar kind of analysis is also used for analyzing the behavior of spin polarization in the system with ${{\varphi }}_{\nu }=\pi \mathrm{/2}$.

In addition to these features it is also observed that for both zero and non-zero values of AAH phase, $P$ gradually decreases with increasing ${t}_{C}$ as two opposite spin electrons are allowed to pass more easily from the source to drain electrode without encountering magnetic region. Thus, from the results presented in Fig. 5, it can be emphasized that *controlling* ${t}_{C}$ *externally, the spin polarization can be varied in a wide range (* $+1$ *to* $-1$ *and vice versa) through this nano-junction, without changing any other physical parameters*. This is indeed an interesting observation and we believe that it can be verified through an experimental setup.

### Regulation of spin polarization by ${{\boldsymbol{\varphi }}}_{{\boldsymbol{v}}}$

**Figure 7:**
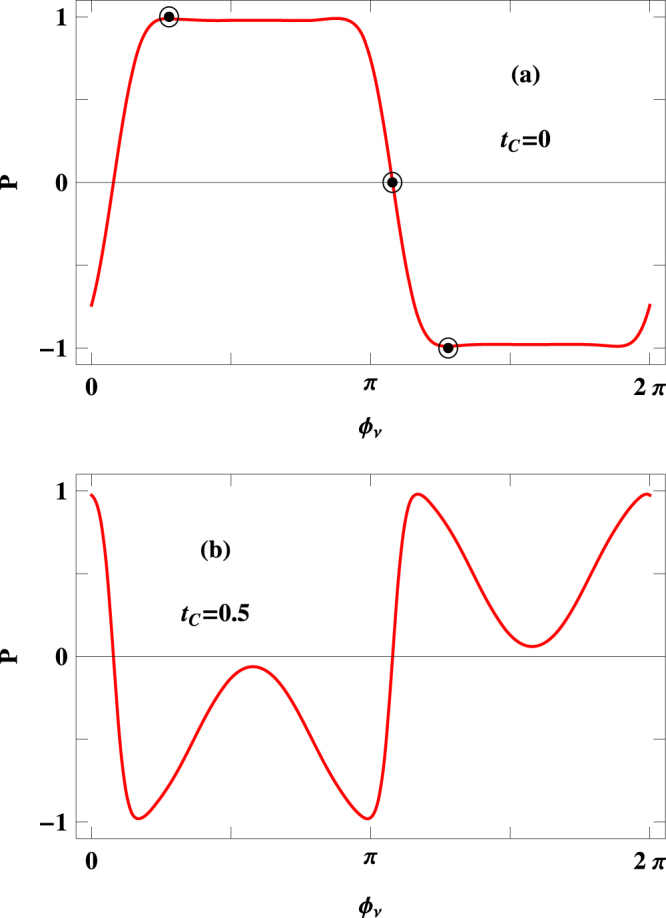
$P$-${{\varphi }}_{\nu }$ characteristics at two typical values of ${t}_{C}$ for a $100$-site ring considering $V=0.25\,$ V.

**Figure 8:**
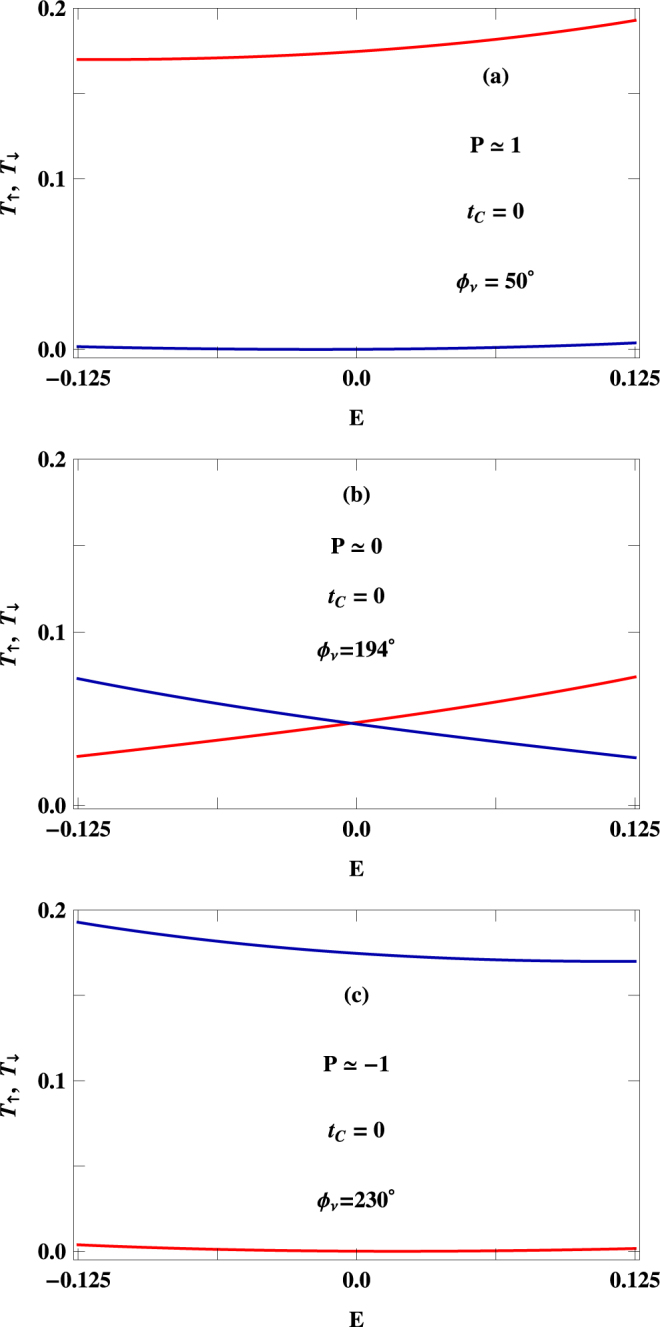
Energy dependence of ${T}_{\uparrow }$ (red curve) and ${T}_{\downarrow }$ (blue curve) at three different values ${{\varphi }}_{\nu }$ those are represented by encircled dots in Fig. 7(a). The other physical parameters are kept constant as taken in Fig. 7.

To establish the specific dependence of $P$ on phase factor ${{\varphi }}_{\nu }$, in Fig. 7 we present the results for a $100$-site ring considering two typical values of ${t}_{C}$. Quite interestingly we see that, like Fig. 5, here also the spin polarization coefficient exhibits a wide range of variation (cent percent up spin polarization to cent percent down spin polarization and vice versa) upon the change of ${{\varphi }}_{\nu }$ for a fixed ${t}_{C}$. The role of ${t}_{C}$ on phase reversal is also clear from the spectra given in Fig. 7(a) and (b). This interesting pattern can be visualized from the transmission spectra placed in Fig. 8, where we present the variations of up and down spin transmission probabilities in a particular energy window associated with the voltage bias, selectively choosing three arbitrary points from the $P$-${{\varphi }}_{\nu }$ curve of Fig. 7(a), represented by encircled dots, where $P$ becomes $\sim +1$, $0$ and −1, respectively. For a particular phase a situation may arise where only up spin electrons transmit resulting *P* = +1, and the other situation can also happen for another phase value where only down spin electrons propagate yielding $P=-1$. The third possibility is that for a specific ${{\varphi }}_{\nu }$ both electrons can contribute equally in a typical voltage window providing vanishing transmission probability. All these possible cases are visualized clearly from Fig. 7. *Since this phase factor* ${{\varphi }}_{\nu }$ *is tuned externally, we can suggest that the present model can be utilized as a phase controlled device for getting selective spin transmission through a nano-junction*.

Like the case of controlling spin polarization by introducing ${t}_{C}$, one may think whether there is any possibility to expect the *wide variation of spin polarization* as a function of phase factor ${{\varphi }}_{\nu }$ without doing any numerical calculations or not. The answer is of course yes, since it depends on which spin channel (up or down) is dominating the other for a specific energy window associated with bias voltage $V$. The widths of up and down spin bands of the magnetic quantum ring essentially depends on the factors ${\varepsilon }_{i}$, $h$ and NNH integral $t$. Based on these parameter values we get an overlap between the two spin bands over a finite energy window, while no overlap is obtained for other energy regions. This overlapping region, on the other hand, can be controlled by tuning the phase factor ${{\varphi }}_{\nu }$ as it eventually regulates the site energy ${\varepsilon }_{i}$ through a cosine modulation term. Thus, for a fixed Fermi energy, when overlap region comes within a voltage window for a specific ${{\varphi }}_{\nu }$, vanishingly small spin polarization is observed, whereas keeping all other parameters unchanged we can shift the overlap region from the voltage window by tuning ${{\varphi }}_{\nu }$ and in that case high degree of up (down) spin polarization is obtained depending on the specific channel. This is exactly what we see in Fig. 7.

It is to be noted that when all site energies (${\varepsilon }_{i}$’s) are same i.e., the system becomes an ordered magnetic ring, the eigenenergies of up and down spin bands can be evaluated analytically so that their overlap can easily be estimated. While, for correlated site energies (like our present model) analytical solution is no longer available. Though we can intuitively estimate the wide variation of spin polarization with phase ${{\varphi }}_{\nu }$ without doing numerical calculations, complete transmission-energy spectrum only reveals the precise determination of spin polarization at different phases.

### Simultaneous variation of $P$ by ${{\boldsymbol{t}}}_{{\boldsymbol{C}}}$ and ${{\boldsymbol{\varphi }}}_{{\boldsymbol{v}}}$

**Figure 9:**
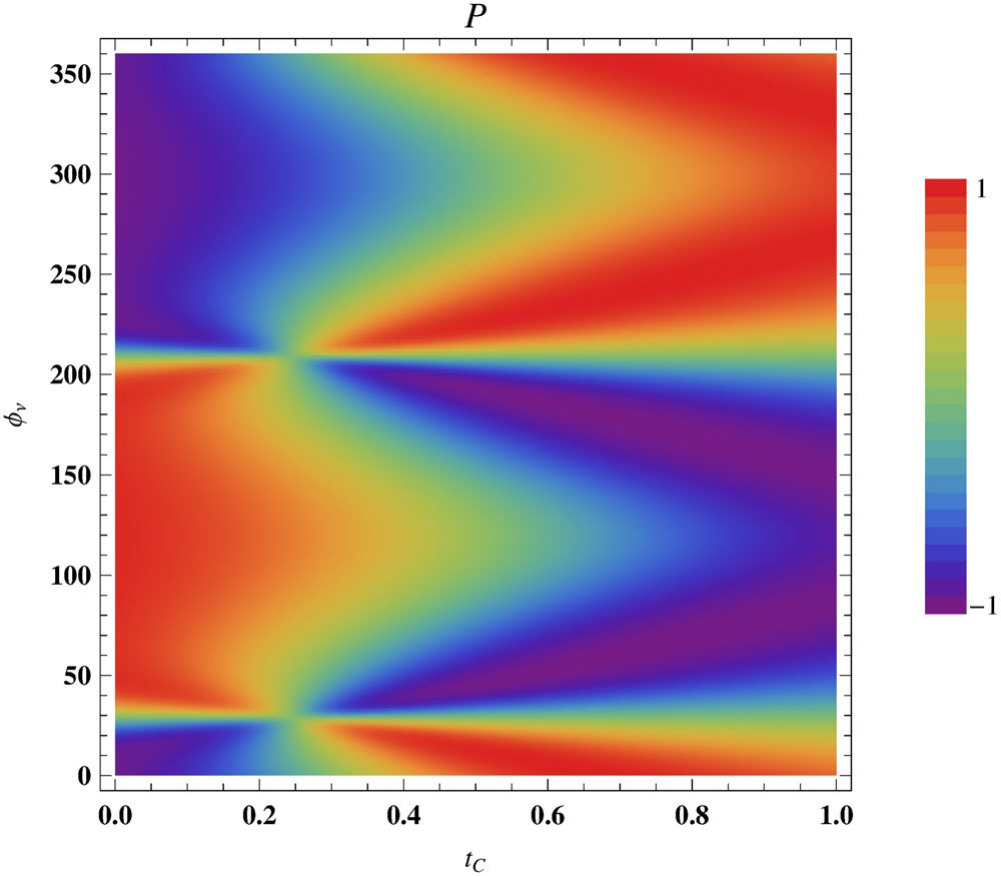
Simultaneous variation of $P$ with ${t}_{C}$ and ${{\varphi }}_{\nu }$ for a $60$-site ring at $V=0.25\,$ V.

From the above analysis (Figs 5–8) naturally the question appears how the spin polarization gets modified with the simultaneous variation of both ${t}_{C}$ and ${{\varphi }}_{\nu }$. The answer is given in Fig. 9 where we present the dependence of $P$ as functions of ${t}_{C}$ and ${{\varphi }}_{\nu }$ considering a $60$-site ring at $0.25\,$ Volts. This is a clear picture to visualize the combined role of these two externally controlling parameters. For lower ${t}_{C}$, $P$ becomes ∼ + 1 or ∼ − 1 for a wide range of ${{\varphi }}_{\nu }$ providing a broad zone of identical color (red or pink), while the width of these zones becomes narrow down as we move towards higher ${t}_{C}$. This diagram suggests that the physical pictures are valid over a large range of parameter values, rather than a specific ${t}_{C}$ and ${{\varphi }}_{\nu }$, which claims the robustness of our observation.

### Spin Inversion

Finally, we concentrate on spin-flip scattering through this nano-junction. To get spin-flip transmission we have to set a non-zero value of ${\theta }_{i}$, as $\theta ={0}^{\circ }$ (we can call ${\theta }_{i}=\theta$ $\forall$ $i$, for simplification) does not involve the factors ${\sigma }_{+}$ and ${\sigma }_{{-}}$ in the spin-flip term ${\overrightarrow{h}}_{i}\mathrm{.}\overrightarrow{\sigma }$ (Eq. 2)^54^ which are responsible for spin flipping.

**Figure 10:**
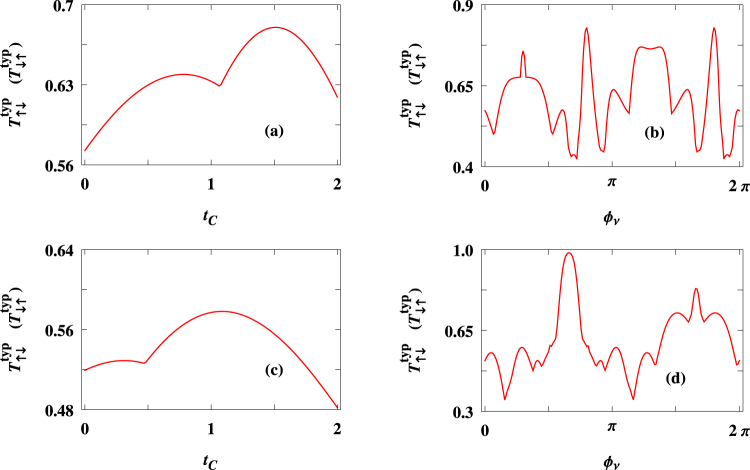
Spin flip transmission probabilities ${T}_{\uparrow \downarrow }^{\,\,typ\,}$ (${T}_{\downarrow \uparrow }^{\,\,typ\,}$) as a function of ${t}_{C}$ (${{\varphi }}_{\nu }$) for two different ring sizes, where the upper and lower rows correspond to $N=60$ and $40$, respectively. Here we choose ${\theta }_{i}=\pi \mathrm{/2}$ $\forall$ $i$. The typical value of spin-flip transmission probability is determined by taking the maximum value of ${T}_{\sigma {\sigma }^{\text{'}}}$ from the ${T}_{\sigma {\sigma }^{\text{'}}}$-$E$ curve considering the variation of $E$ within the energy window $-4\le E\le 4$.

In Fig. 10 we present the spin-flip transmission probabilities ${T}_{\uparrow \downarrow }^{\,\,typ\,}$ (${T}_{\downarrow \uparrow }^{\,\,typ\,}$) for two different ring sizes considering $\theta =\pi \mathrm{/2}$, where the upper and lower rows correspond to $N=60$ and $40$, respectively. The typical value of spin-flip transmission probability is determined by taking the maximum value of ${T}_{\sigma \sigma \text{'}}$ from the ${T}_{\sigma \sigma \text{'}}$-$E$ curve considering the variation of $E$ within the energy window $-4\le E\le 4$. From the spectra it is observed that for a finite (small) window of AAH phase a complete spin reversal takes place (see Fig. 10(d)), while in other cases though full spin inversion is not available but the degree of spin inversion is sufficiently high at some particular ${t}_{C}$ and ${{\varphi }}_{\nu }$ windows (Fig. 10). It indicates that by controlling the physical parameters a possibility may arise to achieve complete spin inversion through this nano-junction.

**Figure 11:**
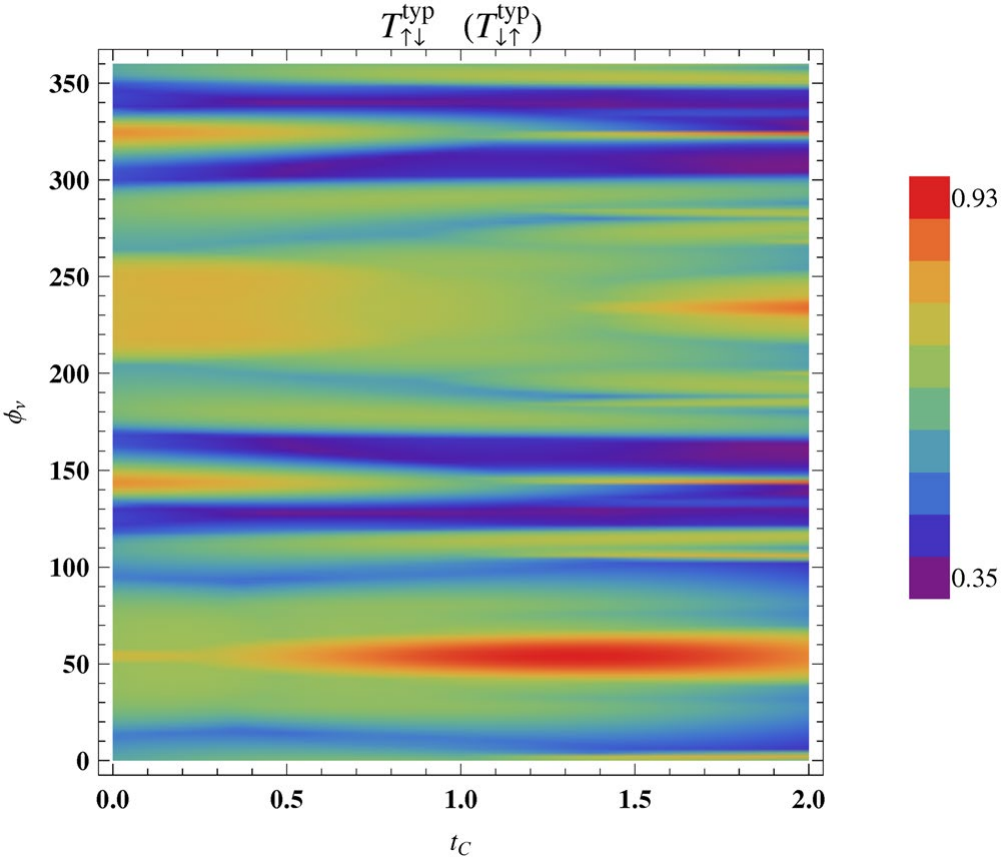
Simultaneous variation of ${T}_{\uparrow \downarrow }^{\,\,typ\,}$ (${T}_{\downarrow \uparrow }^{\,\,typ\,}$) with ${t}_{C}$ and ${{\varphi }}_{\nu }$ for a $60$-site ring considering ${\theta }_{i}=\pi \mathrm{/2}$ $\forall$ $i$. ${T}_{\sigma \sigma \text{'}}$ is determined in the same way like Fig. 10.

To make it more clear in Fig. 11 we present ${T}_{\uparrow \downarrow }^{\,\,typ\,}$ (${T}_{\downarrow \uparrow }^{\,\,typ\,}$) as functions of both ${{\varphi }}_{\nu }$ and ${t}_{C}$ considering $N=60$ and $\theta =\pi \mathrm{/2}$. Almost $95\, \%$ spin inversion takes place for a reasonable window of the parameter values which definitely suggests an experimental verification as the results are not so sensitive with fine tuning of these parameters. In addition, we would like to state that though the results presented in Fig. 11 are computed for a specific value of $\theta$, almost similar kind of physical picture (viz, large degree of spin polarization for wide window of parameter values) is also obtained for other values of $\theta$. Therefore, we do not repeat the same thing considering different values of this parameter ($\theta$).

### Experimental Perspective

**Figure 12:**
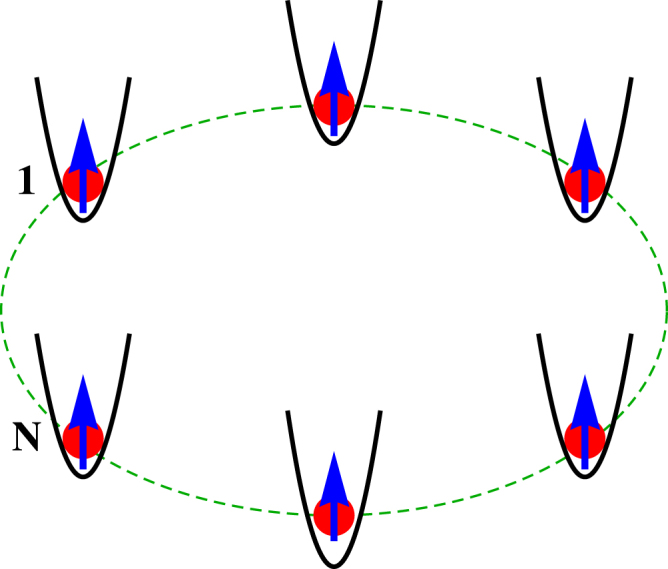
Schematic view of a ring-shaped geometry where trapping potentials are formed by two laser beams. In each such potentials (described by black line) a magnetic atom is trapped to form a magnetic quantum ring with modulation in site energy. The source and drain electrodes will be connected at the sites $1$ and $N$, respectively.

In order to substantiate the proposed scheme of tuning spin polarization via controlling the phase factor ${{\varphi }}_{\nu }$ in laboratory we have to think about the possible realization of an experimental setup. Our essential goal is to develop a $1$ D magnetic quantum ring where site energies are modulated in the form of standard AAH model i.e., in one hand the site energies are quasiperiodic and in the other hand this deterministic energy profile can be regulated externally (which is ${{\varphi }}_{\nu }$ in our model). Several experimental proposals have been made along this direction to construct such a ring-like geometry, in fact different other geometrical shapes can also be designed^55–59^. Two counter propagating laser beams having wave vectors ${k}_{1}$ and ${k}_{2}$ are used for generating such a quasiperiodic potential, where the incommensuration parameter is defined by the factor ${k}_{1}/{k}_{2}$. Once the profile is formed by optical means then magnetic atoms are trapped in the dip regions as shown in Fig. 12. Tuning any one the two laser beams the profile can be regulated which practically describes the change of phase factor ${{\varphi }}_{\nu }$ externally. Thus, a magnetic quantum ring with finite modulation in site energies can be formed through which spin-dependent transport can be tested. The details of experimental realization are available in refs^55–59^ Before we end, we would like to point out that with the help of interfering laser beams different kinds of aperiodic lattices (our model is one such case) can be formed, but it is very hard to design a setup to map a random disordered model since in this case site energies are no longer correlated.

The other scheme of spin current regulation by means of tuning ${t}_{C}$ can easily be implemented in a laboratory setup. One can do it either by changing the separation between the source and drain electrodes or by rotating them^49^.

## Summary

To conclude, in the present work *two new mechanisms* have been pointed out for the regulation of spin polarization as well as spin inversion through a magnetic nano-junction. A complete sign reversal of spin polarization (i.e., *P* = +1 to *P* = −1 and vice versa) takes place by changing any one of the two controlling parameters (viz, ${{\varphi }}_{\nu }$ and ${t}_{C}$). The tunneling coupling ${t}_{C}$ between the electrodes can be regulated *externally* by some mechanical ways, and the other physical parameter i.e., AAH phase ${{\varphi }}_{\nu }$ can also be tuned *externally*. Our results are valid for a wide range of parameter values, and thus, definitely an experimental verification can be made along this line. Focusing in that direction, finally we have discussed briefly how the proposed model can be realized in laboratory.

We have given a detailed theoretical description for the calculation of spin dependent transmission probabilities based on quantum wave-guide theory which might be helpful for investigating spin dependent transport through any such magnetic system. The scattering theory presented here is the extension of earlier studies where spin degrees of freedom have been ignored. So, in that context our theoretical prescription based on wave-guide theory involving electron spin is quite new, to the best of our knowledge.

In our forthcoming work we will analyze the behavior of spin polarization in such a nano-junction where two different phases, namely ${{\varphi }}_{\nu }$ and ${{\varphi }}_{\lambda }$, are introduced in site potentials and hopping integrals, respectively, along with the external tunneling coupling ${t}_{C}$. Both these phases (${{\varphi }}_{\nu }$ and ${{\varphi }}_{\lambda }$) can be regulated simultaneously and independently through an experimental setup, and we strongly believe that some interesting features will be obtained that can be utilized in designing spin based quantum devices.

## Some Additional Points

Here we would like to discuss some additional points for the sake of completeness and the benefit of interested researchers.

A. In our model we have considered identical strength of all magnetic moments (i.e., ${h}_{i}=h$ (say) for all $i$). One can in principle consider different ${h}_{i}$ which means different magnetic sites in the ring. The main reason of not considering different ${h}_{i}$ is that here we intend to focus on the interplay between correlated diagonal disorder (that can be designed experimentally) and the external coupling (shunting path) term ${t}_{C}$. So there are two factors (i) phase in site potential and (ii) ${t}_{C}$, that can be used to regulate spin transmission through the conducting junction. Introduction of different ${h}_{i}$ does not provide any new physical signature. Only the height of the transmission peaks get reduced without changing the polarization characteristics. The same argument also goes to select the other two parameter values (${\theta }_{i}$ and ${{\phi }}_{i}$).
B. In describing the Hamiltonian of the magnetic quantum ring (Eq. 2) we have ignored exchange interaction term between local magnetic moments. So one may ask why we have not considered the exchange term. The reason is that at low temperature this interaction term has very minor impact and does not make any qualitative difference. And the other important point is that since thermal broadening of energy levels is too weak compared to the energy level broadening caused by ring-to-electrode coupling, even moderate temperature are expected to have a very little impact on our qualitative predictions^52^. Therefore only zero temperature has been considered here. Naturally at zero temperature we can ignore this interaction term.
C. It is well known that Rashba SO coupling is responsible for spin-flip scattering. So the question naturally comes can we expect similar kind of characteristic features, as discussed above, if we replace the magnetic quantum ring by a Rashba ring. The answer is of course no. The first thing is that in a two-terminal system only SO coupling is not responsible for producing polarized spin currents. We have to apply a magnetic field to break the Kramer’s degeneracy, and confining of a magnetic field in a small sized ring is always a difficult task. This part has already been discussed in the introduction.
  The other point is that it is very hard to design a Rashba ring considering such a deterministic disordered potential in experiment, whereas magnetic atomic sites can easily be trapped optically. The Rashba term appears because of the asymmetry in the confining potential. So the mechanism is completely different and we do not know whether it is at all possible to design a Rashba ring by constructing a potential profile with the help of two interfering laser beams. May be a theoretical analysis can be done using a two-terminal Rashba ring in presence of magnetic field or considering a three-terminal Rashba ring (where magnetic field is no longer required to get spin polarization in outgoing leads) by this same prescription, but question may arise how to design such a model experimentally.
D. Throughout the numerical analysis we set a specific parameter values of $w$, ${t}_{S}$, ${t}_{D}$ and $t$. Naturally the question may arise how the results get modified if we choose other set of parameter values, for example, if we increase or decrease $w$, ${t}_{S}$, ${t}_{D}$ compared to $t$.

First consider the effect of $w$ and (say) we are increasing $w$. It ($w$) measures the correlated disorder strength. So keeping all other parameters fixed if we increase $w$ then disorder strength will be increased which means electronic states will be less conducting, as expected in correlated disordered systems. Accordingly peak heights in transmission spectra get reduced. So eventually for large enough disorder strength ($w\gg t$) all states of the ring will be almost localized. Under this situation electrons will not enter into the ring geometry. But due to the additional shunting path, which is incorporated by considering a coupling between two electrodes, electron can easily hop from source to drain, avoiding the localized regime i.e., the ring geometry. As the electrons are not entering into the ring they will not experience any spin-dependent scattering and hence for this large enough $w$ we will not get any spin polarization.

We can also think the above situation in other way. Suppose we fix $w$ which is not so large to localize electrons. Under this situation if we increase the coupling term ${t}_{C}$ then electrons will try to pass directly from source to drain, ignoring the ring geometry. In that case also we get decreasing spin polarization. Since disorder effect is well known we do not want to repeat this, whereas we present our results by changing ${t}_{C}$ which on the other hand can be realized in experiments quite easily.

Now we discuss the case where $w$ gets decreased. In this case electron will try to move through the ring, and there are two possible paths in the ring. So in total three possible paths: two arms in the ring (say upper and lower arms) and the third one is the shunting path. Thus combined interference effect will be there which again analogous to the change of ${t}_{C}$ for a fixed $w$. Because of this, we have elaborately described the effect of coupling ${t}_{C}$.

Finally, we focus on the ring-to-electrode coupling effect i.e., how the results get affected by changing ${t}_{S}$ and ${t}_{D}$ with respect to $t$. This coupling effect has already been studied in a series of papers by us and other few authors too. Therefore, we do not want to repeat this behavior once again, and one can easily follow this effect from the refs^31,48,60–62^.
